# Mitochondria in Ischemic Stroke: New Insight and Implications

**DOI:** 10.14336/AD.2017.1126

**Published:** 2018-10-01

**Authors:** Fan Liu, Jianfei Lu, Anatol Manaenko, Junjia Tang, Qin Hu

**Affiliations:** ^1^Discipline of Neuroscience, Department of Anatomy and Physiology, Shanghai Jiao Tong University School of Medicine, Shanghai, China; ^2^Departments of Neurology, University of Erlangen-Nuremberg, Erlangen, Germany; ^3^Department of neurosurgery, Shanghai General Hospital, Shanghai Jiaotong University, School of Medicine, Shanghai, China

**Keywords:** Ischemic stroke, mitochondrial quality control, mitophagy, mitochondrial transfer, neuroprotection

## Abstract

Stroke is the leading cause of death and adult disability worldwide. Mitochondrial dysfunction has been regarded as one of the hallmarks of ischemia/reperfusion (I/R) induced neuronal death. Maintaining the function of mitochondria is crucial in promoting neuron survival and neurological improvement. In this article, we review current progress regarding the roles of mitochondria in the pathological process of cerebral I/R injury. In particular, we emphasize on the most critical mechanisms responsible for mitochondrial quality control, as well as the recent findings on mitochondrial transfer in acute stroke. We highlight the potential of mitochondria as therapeutic targets for stroke treatment and provide valuable insights for clinical strategies.

## 1. Introduction

Stroke is one of the leading causes of morbidity and mortality worldwide. More than 80% of strokes are ischemic and are caused by obstruction of one or more cerebral arteries [1, 2]. Lack of blood supply deprives the brain cells of necessary glucose and oxygen, and disturbs cellular homeostasis, culminating in neuronal death. Currently, tissue plasminogen activator (tPA) is the only effective pharmacological therapy approved by the Food and Drug Administration for acute ischemic stroke since 1996, but its use remains limited due to the narrow therapeutic window [3-6]. It is imperative to develop novel therapeutic strategies of stroke [7, 8].

Mitochondria, as the powerhouse of cell, play a critical role in cell energy homeostasis and are thus inevitably involved in the ischemic neuronal death. After ischemia, due to the reduced blood supply, the energy balance is disrupted and adenosine triphosphate (ATP) synthesis is disturbed. In addition to their fundamental role in energy generation, mitochondria are critical involved in the regulation of such forms of cell death as apoptosis, autophagy and necroptosis. Mitochondrial dysfunction has been regarded as one of the hallmarks of ischemia/reperfusion (I/R) injury which induces neuronal death[9]. Accumulating evidences indicate that the maintaining of the mitochondrial function is crucial for neuron survival and neurological improvement. Therefore, targeting mitochondria is one of the promising neuroprotective strategies for stroke treatment [10]. Recently, it has been demonstrated that mitochondria can be transferred from astrocytes to neurons and served as “help-me signaling” in cell-to-cell communication after cerebral ischemia [11]. In this review, we will discuss current knowledge on the role of mitochondria in cell death and cell survival, and highlight the advance of mitochondria-based therapy in stroke. In particular, we will emphasize the most critical mechanisms responsible for mitochondrial quality control, as well as the recent findings on mitochondrial transfer in acute ischemic stroke.

## 2. Pathophysiology of ischemic stroke

**Figure 1.**
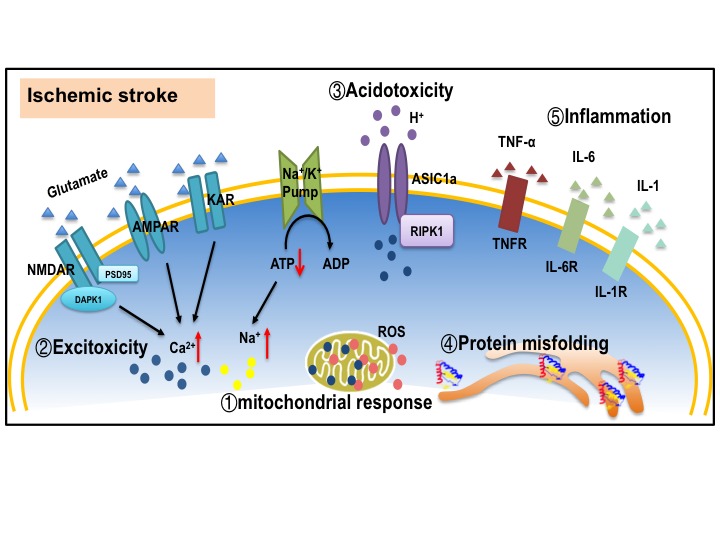
Mechanisms underlying neuronal death in ischemic stroke (1) Mitochondrial response, including excessive ROS production, mitochondrial calcium overloading, and disrupted mitochondria quality control. (2) Excitotoxicity. Excessive glutamate release and impeded reuptake of excitatory amino acids result in the activation of NMDARs, AMPARs and KARs. (3) Acidotoxity. Extracellular acidification leads to ischemic neuronal death by activating acid-sensing ion channel 1a (ASIC1a). (4) Protein misfolding. Protein misfolding and aggregation are observed after brain ischemia. (5) Inflammatory reaction. Microglia are activated and release cytokines and chemokines to induce inflammation reaction. All the factors mentioned above work synergistically to trigger cell death pathways such as apoptosis, necroptosis and autophagy. ROS: reactive oxygen species; AMPAR: α-amino-3-hydroxy-5-methyl-4-isoxazole-propionic acid receptor; NMDAR: N-methyl-D-aspartate receptor; KAR: kainite receptor; DAPK1: death associated protein kinase 1; PSD95: postsynaptic density protein 95; ASIC1a: acid-sensing ion channel 1a; RIPK1: receptor interacting protein kinase 1; TNF-α: tumor necrosis factor-α; IL-6: Interleukin 6; IL-1: Interleukin 1.

Ischemic stroke is a consequence of a critical reduction of regional cerebral blood flow (rCBF), leading to severe oxygen and glucose deprivation. Mitochondrial disfunction induced by oxygen and glucose deprivation occurs within minutes after ischemia, resulting in depletion of ATP production and overproduction of reactive oxidative species (ROS). Compared to other brain cells, neurons have higher energy demand, but their energy reserves are limited. Depletion of ATP is one of the major initiator, which triggers the ischemic cascades such as, membrane ion pump failure, efflux of cellular potassium, influx of sodium, chloride and water, and membrane depolarization [12-14]. Multiple mechanisms, including excitotoxicity, mitochondrial response, free radical release, acidotoxity, protein misfolding, and inflammation have been extensively studied as the events leading to the cellular death and neuronal loss after stroke [15-17], which are depicted in Fig. 1.

**Table 1:** Mitochondrial dynamic-related proteins.

Mammals	Yeast	Role in mitochondria dynamics	Location
Drp1	Dnm1p	Fisson	cytosolic, and recruited to outer membrane during fission
Fis1	Fis1p	Fisson	Outer mitochondrial membrane
Endophilin B1	-	Fisson	cytosolic, and recruited to outer membrane during fission
Mfn1/2	F201p	Fusion	Outer mitochondrial membrane
Opa1	Mgm1p	Fusion	Inner mitochondrial membrane
KIFs	_	Transport	Cytosolic
Cytoplasmic dynein	Dynclil	Transport	Cytosolic

There are two major zones of injury in ischemic brain: the infarct core and the ischemic penumbra. The “infarct core” is the area with the blood flow decline below the threshold of energy failure (15%-20%). The “infarct core” will be irreversibly damaged during few minutes after stroke and cannot be salvaged [18]. On the contrary, ischemic penumbra is the region with impaired functions, but preserved structure integrity [19]. Cells in penumbra are salvageable if reperfusion is established during the early hours or collateral circulation is adequate to maintain the neuronal demand for oxygen and glucose [15]. Therefore, the penumbra is the pharmacological target for the treatment of acute ischemic stroke.

**Figure 2.**
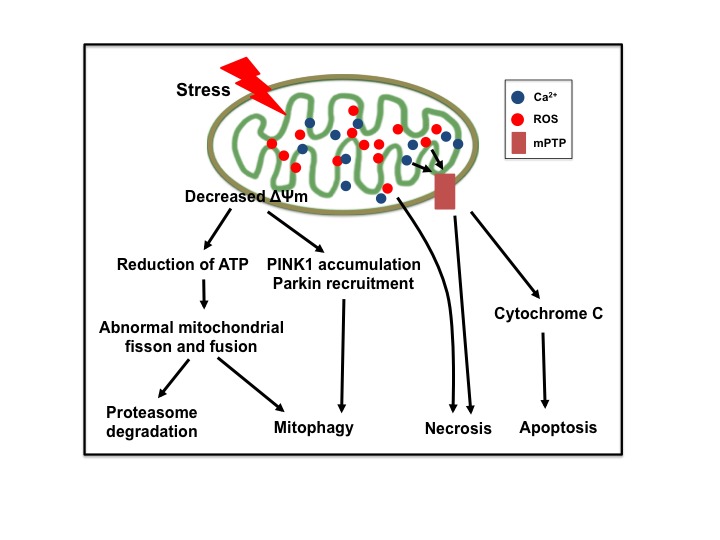
Mitochondria play a central role in ischemic neuronal death Ischemia triggers the depolarization of mitochondrial membrane potential (ΔΨm), reduction of ATP production, accumulation of PINK1, recruitment of Parkin, overproduction of reactive oxygen species (ROS), overloading of matrix calcium, and opening of mitochondrial permeability transition pore (mPTP), eventually leading to neuronal death.

In the infarct core, neuronal cells undergo necrotic alterations, which are accompanied by glutamate release and excitotoxic cell damage to neighboring regions [20]. The penumbra is moderately hypoperfused and energy metabolism is partially preserved, which transiently sustain tissue viability. Timely interventions are effective to avoid the progression of the penumbra into infarction. The molecular mechanisms leading to the cell death in the penumbra differ from those in the infarct area. Several types of regulated cell death such as autophagy, apoptosis, necroptosis, and pyroptosis can be triggered by ischemia and contribute to the post stroke brain injury. To some extent, different kind of cell death can cross-regulate each other [21, 22]. Mitochondria can be considered as one of the master regulators of stress signaling [23-26] (Fig. 2). The impairment of mitochondrial respiratory function and membrane potential activate the cascade of events that leads to neuronal death after ischemia. Depolarized mitochondria initiate excessive ROS production, decreased ATP generation, PTEN-induced putative kinase 1 (PINK1) accumulation, as well as unfolded protein response (UPR) [27]. The increased levels of ROS and overloading calcium open the membrane permeability transition pore allowing for the release of cytochrome c, which activates the effector caspases and the final execution of apoptotic death [28]. PINK1 selectively recruits Parkin and phosphorylates both Parkin and ubiquitin to trigger mitophagy [29]. Interruption of the signaling pathways is apparently able to disrupt, or in some instances greatly delay, the spiral of increasingly vicious reactions that culminate in cell death.

## 3. Structure and function of mitochondria

Mitochondria are ovoid or rod-shaped organelles with double-membrane structure, which consist of four distinct compartments - the outer membrane, the intermembrane space, the inner membrane, and the matrix [30]. The outer membrane contains a number of pores which form large aqueous channels to permit free diffusion of molecules less than 5000 daltons into the intermembrane space. The intermembrane space contains proteins (e.g. cytochrome c) that play major roles in mitochondrial energetics and apoptosis. In contrast to the outer membrane, the inner membrane has much more restricted permeability, equipped with a variety of ion channels and transporters as well as mitochondrial enzyme systems like the electron transport chain. The matrix contains most of the enzymes that responsible for the citric acid cycle reactions. The size, shape and number of mitochondria vary widely depending on the functional state of the cells [31, 32]. And structural reorganization does occur in cases of stress [33, 34].

Mitochondria are pivotal regulators of cell survival. The critical role of mitochondria in cell survival is mainly reflected in 3 aspects. (1) Mitochondria are the hubs of cellular calcium signaling [23]; (2) The enzyme complexes located on the mitochondrial inner membrane are essential components for the maintenance of cell energy requirements and metabolic homeostasis [24]; and (3) Cell survival greatly relies on the integrity and functionality of mitochondria, hence dynamic mitochondrial changes in their shapes and populations are critical for mitochondrial quality control [25, 26]. Neurons are particularly dependent on mitochondria for calcium signaling and ATP production, which makes them more sensitive to ischemia and hypoxia during stroke. As a consequence, mitochondrial defects have much devastating effects in central nervous system (CNS).

## 4. Mitochondrial quality control systems and ischemic stroke

### 4.1 Mitochondria quality control systems

Mitochondria control virtually every aspect of cell function, including managing redox status, modulating Ca^2+^ homeostasis, generating ATP, and regulating response to cellular and environmental stresses. Over the last decade, mitochondria have moved to the forefront of cell biology research for their central role in regulation of cell death signaling. In stroke, abnormalities in mitochondrial membrane potential and ROS over-production induce neuronal injury (Fig. 2). Here we focus on the most recent findings on the role of mitochondrial quality control processes especially regulation of dynamics and mitophagy in stroke, highlighting their potential as therapeutic targets.

Neuronal survival critically depends on the integrity and functionality of mitochondria. A hierarchical system of cellular surveillance mechanisms protects mitochondria against stress and damage, hence ensures the selective removal of dysfunctional mitochondrial proteins. There are 3 levels of well-conserved mitochondria quality control mechanisms [35]. The first level of defense involves a multilayer network of detoxifying systems capable of fighting oxygen- and aldehyde-mediated mitochondrial toxicity. The second level of defense relies to mitochondrial proteases and chaperones responsible for the maintenance of mitochondrial proteostasis. Toxic folding intermediates or the accumulation of aggregates are deleterious for the cells and it is imperative to correct protein folding and maintain cellular homeostasis. The third level of defense involves the control of mitochondrial morphology, quantity of mitochondria through mitochondrial dynamics (fusion and fission) and mitophagy (mitochondrial clearance). Maintaining mitochondrial integrity and elimination of damaged mitochondria are important avenues to preventing widespread mitochondrial dysfunction, oxidative stress, and cell death in ischemic insult. Hence, mitochondrial dynamics and mitophagy play critical roles in maintaining cellular homeostasis and function. Here we will focus on the third level of defense in response to ischemic stress.

### 4.2 Mitochondrial dynamics and ischemic stroke

**Figure 3.**
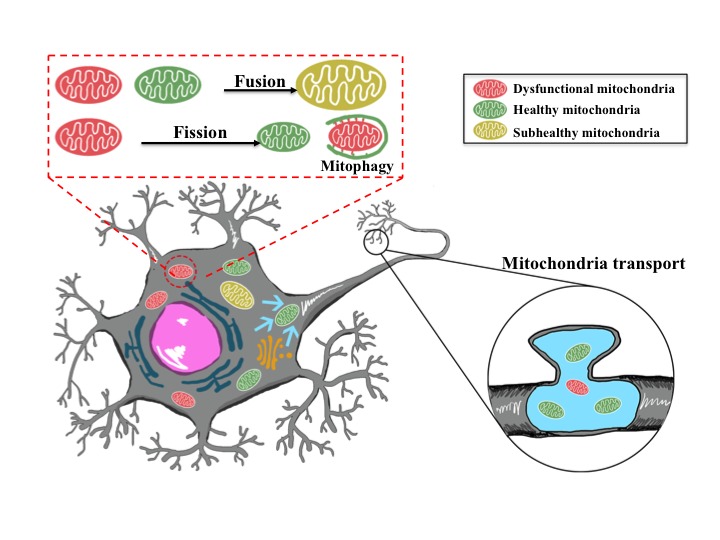
Mitochondrial fusion, fission or transport in neurons Damaged mitochondria can be repaired through fusion with healthy mitochondria, and mitochondrial fission enables the segregation of damaged mitochondria and subsequent elimination via mitophagy. Mitochondria are transported and packed at axonal synapses, and are essential for neuronal transmission and plasticity.

Mitochondria are highly dynamic cellular organelles characterized by their ability to change size, shape and location through highly coordinated processes of fission (separation of a single mitochondrion into two or more daughter organelles), fusion (the opposing reaction) and transport to strategic locations [36-38]. Healthy mature neurons with established synaptic connections exhibited longer, more stationary mitochondria whereas impaired or immature neurons have smaller, more motile mitochondria. Mitochondrial fusion and fission were first observed in yeast in 1994. By now it is known that these processes play critical roles in maintaining mitochondria integrity and function of cells when experiencing metabolic or environmental stresses [39]. Mitochondrial fusion allows the rapid exchange of mitochondrial membranes, mitochondrial DNA (mtDNA), and mitochondrial metabolites within a mitochondrial network; while damaged mitochondria can be repaired through fusion with healthy mitochondria for integrating of contents and promotes cell survival by complementation. On the other hand, mitochondrial fission enables the segregation of damaged mitochondria and leads to their subsequent elimination via mitophagy [40]. Fission and fusion are active processes which require many specialized large guanosine triphosphatases (GTPases). Dynamin-related protein 1 (Drp1), Fis1and Endophilin B1 are required for mitochondrial fission in mammals; Mitofusin 1 (Mfn1), Mitofusin 2 (Mfn2) and Opa1are responsible for fusion. Kinesin superfamily proteins (KIFs) and cytoplasmic dynein are the main microtubule-based motor proteins ensure targeted trafficking of mitochondria and precise regulation of their mobility [41] (Table 1). In neurons, mitochondrial fission is essential for mitochondrial transport to their potential docking sites in axons and dendrites. Defects in mitochondrial fusion, fission or transport lead to impaired mitochondrial motility and function in neurons (Fig. 3). In neuroglial cells, alteration in mitochondrial dynamics contribute to the differential functions of reactive microglia in neurological diseases [42, 43]; and in astrocytes, proinflammatory stimuli induced mitochondrial fission, and ultimately bioenergetics to maintain mitochondrial network [44].

Previously the molecular machineries of mitochondrial fission and fusion were studied separately. Currently, more and more researchers are interested in investigating the coordination/balance of mitochondria dynamics. Mitochondrial fission protein Drp1 was observed to affect the activity of fusion. Santel’s group found that the depletion of Drp1 resulted in reduction of Mfn1, Mfn2 and Opa1 in Hela and HUVEC cells [45]. Consistent with their work, Saita et al reported that loss of Drp1 led to the degradation of Mfns via the ubiquitin-proteasome system [46]. These studies indicated that in addition to the canonical roles of Drp1 in mitochondrial fission, it also acts as regulatory factors that control mitochondrial fusion. Furthermore, Anand et al reported that expression of mitochondrial fusion protein Opa1 unexpectedly triggered mitochondrial fragmentation in Yme1l^-/-^ cells and HEK293T cells, which suggests that mitochondrial fusion protein Opa1 is associated with fission [47]. The coordination of fusion proteins and fission proteins are responsible for fine-tuning mitochondrial dynamics and cell sensitivity to stress.

It is well documented that in ischemic brain injury mitochondrial dynamics are closely linked to their morphology and functions. Mitochondrial fission is an early event required for ischemic neuronal death, which is mediated by Drp1 and Mfn1/2 [48-50]. In primary cultured cortical neurons, nitric oxide exposure triggered dose-dependent mitochondrial fission and caused ultrastructural damage, which are associated with bioenergetic failure and increased ROS production. After middle cerebral artery occlusion (MCAO) in mice, mitochondrial fission occurs as early as 3 h after reperfusion [48]. In the ischemic penumbra, the protein level of both Drp1 and Opa1 increased at 2 days after MCAO, and returned to the baseline at day 7. In the ischemic core, however, the expression of Drp1 and Opa1 progressive decreased until 2 days after transient MCAO [51]. Hyperglycemia further enhanced mitochondrial fission by increasing protein levels of Drp1 and Fis1, as well as decreasing the level of Opa1 in MCAO mice [52]. By contrast, the expression of mitochondrial fusion protein Mfn2 decreased in both *in vivo* and *in vitro* ischemic models, and reduced Mfn2 expression led to mitochondrial dysfunction and disruption of Ca^2+^ homeostasis [49, 50]. Using transgenic mice expressing mitochondria targeted yellow fluorescent protein (mito-eYFP), Owens and his colleagues examined cell-type specific changes of mitochondrial dynamics in hippocampal region following global cerebral ischemia in mice [53]. In cornuammonis 1 (CA1) area of hippocampus, mitochondrial fragmentation started as early as 2 h after the reperfusion and reached the peak at 24 h. Similar observations were done in the CA3 and dentate gyrus (DG) area; however, mitochondria in CA3 and DG neurons started to refuse after 24 h of reperfusion; Astrocytes underwent transient mitochondrial fission from 2 h after ischemia and regained their normal shape at 24 h of recovery [53]. Their data first suggested that ischemia resistant neurons were able to shift their mitochondrial dynamics toward fusion after extensive fragmentation. Recently, using *in vivo* two-photon microscopy, Kislin et al visualized the fragmentation of neuronal mitochondria in the living mouse brain [54]. They induced severe global ischemia by bilateral common carotid artery occlusion, furthermore they induced severe focal stroke injury by Rose Bengal photosensitization and moderate and mild traumatic brain injury by focal laser lesion or mild photo-damage respectively. Mild injury induced transient mitochondrial fragmentation and decreased dendritic spine density, however, severe brain ischemia caused mitochondrial fragmentation and dendritic damage. Their findings showed that alterations of mitochondrial morphology were very sensitive to ischemia and were reversible in moderate and mild injury [54].

**Table 2:** Chemical agents targeting mitochondrial dynamics in ischemic stroke

Agents	Proposed mechanisms	Refs.
Drp1 siRNA	Inhibition of fission via DRP1 down-regulation	[55-57, 61]
Drp1 cysteine mutation	Inhibition of fission by preventing S-nitrosylation induced Drp1 activation	[59]
Ginkgolide K	Inhibition of fission by increasing Drp1 phosphorylation and inhibiting Drp1 recruitment	[60]
mdivi-1	Inhibition of fission by inhibiting the GTPase activity of Drp1	[55, 61]
P110	Inhibition of fission by inhibiting Drp1 enzyme activity and blocking Drp1/Fis1 interaction	[62, 63]
Mfn2 overexpression	Promotion of fusion	[49, 50]

Pharmacological agents or genetic interventions that either inhibiting fission or promoting fusion generally protect neurons from ischemic injury (Table 2). Down-regulation or inhibition of Drp1 reduced mitochondrial fission and maintained normal mitochondrial morphology and function in ischemic neurons [55-57]. Drp1 siRNA and small molecule inhibitors of Drp1 prevented mitochondrial fission, loss of mitochondrial membrane potential, and cell death in glutamate toxicity or OGD *in vitro*, and after ischemic brain damage *in vivo* [57]. However, Drp1 knockout caused embryonic lethality in mice [58]. Drp1 was activated by S-nitrosylation and this process can be prevented by cysteine mutation to protect mitochondria from fragmentation [59]. Ginkgolide K, a natural compound found in Ginkgo biloba leaves, attenuated neuronal apoptosis in OGD model by increasing Drp1 phosphorylation at Ser637 and inhibiting Drp1 recruitment [60]. Mitochondrial division inhibitor mdivi-1, a selective inhibitor of mitochondrial fission protein Drp1, protected brain from OGD reperfusion injury and MCAO through suppressing the ROS production and decreasing the expression of cytochrome C [55, 61]. P110, a novel and selective peptide inhibitor of mitochondrial fission, inhibited Drp1 enzyme activity and blocked Drp1/Fis1 interaction [62]. It abolished Drp1-dependent p53 stabilization on mitochondria and reduced brain infraction in rats subjected to brain ischaemia/reperfusion injury [63]. Promoting mitochondrial fusion also protects cell from injury. Mfn2 overexpression attenuated mitochondrial dysfunction and restored mitochondrial morphology [49, 50]. Interestingly, treadmill exercise pretreatment enhanced mitochondrial fusion via up-regulation of Opa1 after cerebral ischemia [64].

### 4.3 Mitophagy and its role in ischemic stroke

Mitophagy was first described by Lemasters and colleagues in 2005, who determined that mitophagy is an autophagic response, which is responsible for specifically removal of damaged mitochondria to maintain mitochondria homeostasis [65]. The molecular mechanisms regulating mitophagy were originally characterized in yeast. Uth1p and Aup1p are two major proteins required for mitophagy in nutrient deprivation or stationary phase, respectively [66, 67]. However, the corresponding mammalian homologues of Uth1p and Aup1p are yet to be identified. The mechanisms of mitophagy in mammalian cells can be generally classified as ubiquitin (Ub)-dependent and Ub-independent pathways. Theserine/threonine kinase PINK1 and the E3 ligase Parkin pathway is currently the best understood Ub-dependent mechanism of mitophagy for the recognition of damaged mitochondria [68].

Recently mitophagy has received increasing attention in stroke and has been evidenced in *in vivo* models and in cultured neurons. In ischemic stroke, mitophagy could be predominantly mediated by PINK1/Parkin pathway. However, the role of mitophagy in the development of ischemic brain injury remains controversial. Accumulating data have showed that mitophagy is a double-edged sword that can be protective or destructive after experimental stroke [69-73]. Most studies support the hypothesis that mitophagy favors neurons adapted to the stress by removing impaired mitochondria and suppressing cell death signaling cascades. These studies proposed that mitophagy is a promising therapeutic target for stroke treatment. In permanent MCAO, mitophagy was triggered at as early as 1 hour and involved in the removal of damaged mitochondria and cellular survival in a Drp1-dependent way [74]. Mitophagy was also activated in the reperfusion phase after MCAO and contributed to the inhibition of post stroke apoptosis [69]. Rapamycin treatment improved mitochondrial function through enhancing mitophagy after experimental ischemic stroke [75]. The neuroprotective role of methylene blue in acute cerebral ischemia can also be explained by promoting mitophagy and maintaining the mitochondrial membrane potential, which consequently decreased necrosis [76]. Melatonin-mediated mitophagy attenuated the early brain injury after subarachnoid hemorrhage through inhibition of NLRP3 inflammasome activation [77]. Mitophagy also contributed to the neuroprotection induced by limb remote ischemic conditioning after ischemic stroke [73]. The destructive role of mitophagy after cerebral ischemia has also been reported [70, 71]. Inhibition of mitophagy by decreasing p-Drp1 and Parkin mediated the neuroprotective effects of carnosine in rats after MCAO[70], and excessive induction of mitophagy leads to cell death in neonatal stroke[71]. The harmful roles of mitophagy in stroke have not been adequately emphasized. Further investigations are required to understand whether mitophagy is beneficial or detrimental after stroke. It appears that the effects of mitophagy depend on the severity of it. While physiological or mild levels of mitophagy favor the neuronal survival, intensive or excessive levels could be lethal and exacerbate the ischemic brain injury.

## 5. Intercellular Mitochondrial transfer

### 5.1 Intercellular mitochondrial transfer and potential mechanisms

For a long time, mitochondria were considered to be retained intracellularly for their lifetime. Recently, intercellular exchange of membrane vesicles and organelles through structures such as tunneling nanotubes (TNT) or microvesicles has been evidenced [78, 79]. Mitochondrial transfer has been observed in several cardiovascular injury models, and has also been demonstrated in experimental stroke [11, 80, 81]. In 2006 the horizontal transfer of mitochondria, from human mesenchymal stem cells (MSCs) to somatic cells *in vivo* was shown by Prockop’s lab [82]. Furthermore, it has been documented that intercellular mitochondrial transfer occurs in different cell types both *in vitro* and *in vivo* [11, 80, 81, 83-87]. MSCs are indicated as the most popular donator for mitochondrial transfer. Many studies suggest that transfer of mitochondria from one cell to another is a protective mechanism accountable for rescue of injured cells from mitochondrial dysfunctions in response to stress [11, 88, 89]. When co-cultured with mitochondria dysfunctional somatic cells, human MSCs developed cytoplasmic projections and steamed mitochondria to rescue the mitochondrial function of the somatic cells [82]. Bone marrow derived stromal cells protected against acute lung injury by restoration of alveolar bioenergetics through Cx43-dependent alveolar attachment and mitochondrial transfer [85]. In I/R injury, MSCs transferred mitochondria to injured endothelial cells through TNT-like structures, and inhibited endothelial cells from apoptosis by rescue of aerobic respiration [81]. All these data corroborate that mitochondrial transfer undertakes metabolic cross-talk between healthy cells and injured cells. The released mitochondria can be taken and reprogrammed by recipients to activate signals for cell survival. Therefore, mitochondrial transfer is greatly expected to be novel therapeutic approaches for mitochondrial diseases or disorders, as well as for stroke.

**Figure 4.**
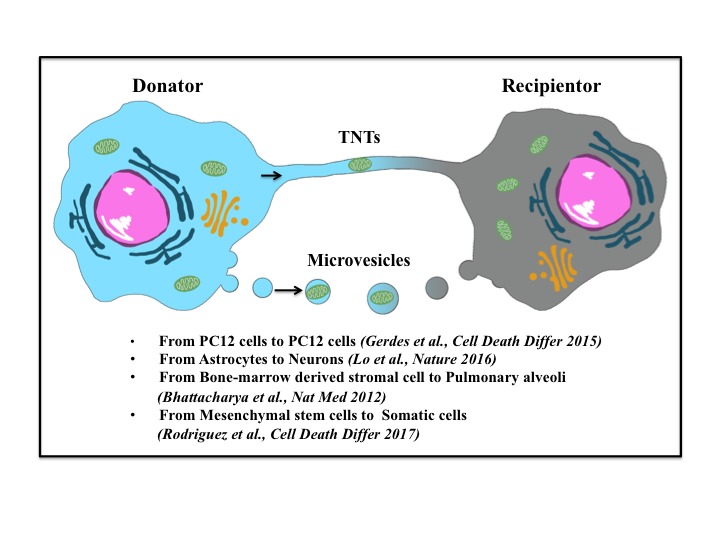
Intercellular mitochondrial transfer Mitochondria can be released by donate cells and uptake by recipient cells. Stressed or dying cells release mitochondria through tunneling nanotubes (TNTs) or microvesicles. Mitochondrial transfer can occur between same or different cell types.

The mechanisms underlying mitochondria release and uptake by recipient cells are still unclear. Stressed cells or dying cells release mitochondria serving as danger warning signals. When stimulated with tumor necrosis factor alpha (TNF-α), mouse embryonic fibroblasts and hepatocytes extruded mitochondria through auto-lysosomal exocytosis [90]. In human T-lymphoblastic leukemia cells and murine fibroblast cells, TNF-α induced necroptosis was accompanied by extracellular release of mitochondria [91]. This is an active process, which occurs before the disruption of the plasma membrane. The mitochondria were intact and no mtDNA was emitted. Several molecular mechanisms have been proposed to regulate intercellular mitochondrial transfer. A study by Ahmad and his colleagues reported that Miro1 promoted mitochondrial transfer from MSCs to epithelial cells, which was well-directed rather than a general exchange of contents [92]. In human acute myeloid leukemia, ROS drives TNTs formation and regulates the transfer of mitochondria from bone marrow stromal cells to leukemia blasts through NADPH oxidase-2 [93]. Astrocytic release of extracellular mitochondria was mediated by a calcium-dependent mechanism involving CD38 and cyclic ADP ribose signaling in ischemic stroke mice, and integrin-mediated Src/Syk signaling might be involved in the endocytosis of mitochondria in neurons [11]. Several other studies suggested that macrophage cells and immune cells recognized the released mitochondria by damage-associated molecular patterns [27, 91, 94]. However, the health status and the fates of the transferred mitochondria before/after internalization were not assessed in these studies. Recently, using mitochondrial reporter mitoROGFP, which is able to detect oxidative stress, Melentijevic et al showed that stressed or damaged mitochondria were preferentially extruded in adult neurons from Caenorhabditis elegans [88]. Human macrophages and dendritic cells engulfed released mitochondria and triggered the secretion of proinflammatory cytokines [91]. In a co-culture system of MSCs with cardiomyocytes or endothelial cells subjected to oxidative challenge with hydrogen peroxide (H_2_O_2_), mitochondria from suffering cells were engulfed and degraded by MSCs, leading to induction of the cytoprotective enzyme heme oxygenase-1 and stimulation of mitochondrial biogenesis [95]. Such studies have revealed that organelles can functions as a new aspect of transcellular signaling like moleculars, which opens an entirely new perspective on cell-to-cell communication (Fig. 4). Elucidation of the mechanisms of mitochondrial transfer will help to establish therapy-based mitochondrial restoration for human diseases caused by mitochondrial dysfunction.

### 5.2 Mitochondrial transfer techniques and its application in ischemic stroke

In 1982, Clark and Shay first transferred mitochondria with antibiotic-resistant genes to sensitive cells, which enabling the cells to survive in a selective medium [96]. Their observations opened the new field of research on mitochondrial transfer. Various approaches have been developed to replenish the damaged mitochondria in recipient cells and suggest new possible applications. Coincubation is the first and simple approach tested and the efficiency differs in different cell lines [96]. Microinjection and such other invasive techniques as nanoblades can quickly repopulate the resident mtDNA, but they are less efficient than coincubation for the limited cell numbers [97, 98]. Additional techniques has been developed to facilitate the mitochondria internalization into the recipient cells, for example, conjugating the mitochondria with cell-penetrating peptide Pep-1, labeling mitochondria with anti-TOM22 magnetic beads, and increase of mitochondrial uptake by MitoCeption technique [99-101]. In the *in vivo* studies, mitochondria were either directly injected into the ischemic heart or delivered intracoronarlly at the onset of reperfusion in rabbits[102, 103]. Both two methods effectively decreased the infarct size and restored the heart functions. In ischemic stroke, direct injection of astrocyte-derived mitochondria into the peri-infarct cortex 3 days after MCAO promoted neuron survival by increasing the level of phosphorylated AKT and BCL-XL [11]. In neurons, mitochondria are concentrated within the presynaptic nerve terminal. Due to the necessity of long-distance transportation mitochondria can not be replenished quickly after ischemia. The transfer of extracellular mitochondria may, therefore, open a new avenue for neuroprotective strategies.

The beneficial effects of mitochondrial transfer in stroke were first studied by Po-Jui Huang and his colleagues [104]. Transferring exogenous mitochondria via in-situ injection or systemic administration of exogenous mitochondria mitigated cell death both in MCAO rats and OGD neurons and improved the motor performance of rats after MCAO [104]. The grafted mitochondria were traced by pre-labeled with BrdU for their distribution and were internalized by neurons, astrocytes and microglia. Disrupting electron transport or ATPase synthase of the donor mitochondria significantly attenuated the protective effect. Their data supported that in-situ injected mitochondria can be taken by neurons, astrocytes and microglia in MCAO mice, and the intact respiratory activity is essential for the mitochondrial potency on neural protection [104]. A followed-up study on mitochondrial transfer in stroke was performed in Eng H. Lo’s group. Cultured astrocytes were observed to produce functional extracellular mitochondria, which are able to increase the ATP levels and viability of neurons suffered from OGD. Extracellular mitochondria injected in the peri-infarct cortex of MCAO mice were found in neurons. The transplantation of mitochondria resulted in an upregulation of cell-survival-related signals in MCAO mice[11]. The authors suggested that astrocytes have the ability to transfer healthy mitochondria to rescue the damaged neurons after stroke. In their another study, extracellular mitochondria were detected in cerebrospinal fluid (CSF) with decreased mitochondrial membrane potentials in subarachnoid hemorrhage (SAH) rats [105]. In clinic, the similar results were found in SAH patients; and higher membrane potentials of mitochondria in the CSF were correlated with good clinical recovery at 3 months after SAH onset [105]. It is possible that after stroke, neuronal cells transfer mitochondria not only for disposal, but also for recycling and to quickly counteract the energy deficits caused by ischemia.

## 6. Conclusions and perspectives

Mitochondrial disfunction is an early and initiating events in the pathophysiology of stroke [106]. Defected bioenergenetics, abnormal mitochondrial morphology and structure, and aberrant mitochondrial dynamics play a central role in the activation of death signaling pathways [107]. Interventions targeting mitochondrial quality control and mitochondrial dynamics by pharmacological agents or genetic modifications have been demonstrated neuroprotective in preclinical studies [49, 108, 109]. Unfortunately, clinical trials using mitochondrial protectants or antioxidants, either alone or in combination with other therapeutic approaches, have been unsuccessful. Targeting mitochondria pharmacologically in the clinic is still challenging. Recently, the evidences of mitochondrial transfer open an entirely new perspective in the intercellular communication. Emerging findings indicate that mitochondria themselves can function as “help-me” signaling in response to diverse extracellular stimulus and recruit adjacent cells to rescue the injured cells. Removing the impaired mitochondria and replenishing healthy mitochondria are promising therapeutic approaches to treat hypoxia/ischemia related diseases, especially in CNS which mitochondria are abundance in the distal axonal synapses and dendritic protrusions [110-112]. Though clinical application of mitochondrial transplantation has been performed successfully in pediatric patients suffered from myocardial ischemia-reperfusion injury recently, challenge still remains to implement mitochondrial transfer in clinic widely and safely [113]. Great efforts need to be devoted to investigating the detailed mechanisms on mitochondria release from donor cells and recognition by recipients, the generalizability of the beneficial results, and the ethical implications related to artificially mitochondria transferred. Addressing these hurdles underlying mitochondrial transfer will develop novel manipulation targets for the diseases related to mitochondrial dysfunction.
